# Genome-Wide Identification and Drought Stress Response Pattern of the NF-Y Gene Family in *Cymbidium sinense*

**DOI:** 10.3390/ijms25053031

**Published:** 2024-03-06

**Authors:** Linying Wang, Xuewei Zhao, Ruiyue Zheng, Ye Huang, Cuili Zhang, Meng-Meng Zhang, Siren Lan, Zhong-Jian Liu

**Affiliations:** 1 Key Laboratory of National Forestry and Grassland Administration for Orchid Conservation and Utilization, College of Landscape Architecture and Art, Fujian Agriculture and Forestry University, Fuzhou 350002, China; 2 College of Forestry, Fujian Agriculture and Forestry University, Fuzhou 350002, China

**Keywords:** *Cymbidium sinense*, NF-Y genes, abiotic stress, drought stress responsiveness, expression analysis

## Abstract

*Cymbidium sinense,* a type of orchid plant, is more drought-resistant and ornamental than other terrestrial orchids. Research has shown that many members of the *NUCLEAR FACTOR Y (NF-Y)* transcription factor family are responsive to plant growth, development, and abiotic stress. However, the mechanism of the *NF-Y* gene family’s response to abiotic stress in orchids has not yet been reported. In this study, phylogenetic analysis allowed for 27 *CsNF-Y* genes to be identified (5 *CsNF-YAs*, 9 *CsNF-YBs*, and 13 *CsNF-YC* subunits), and the *CsNF-Y*s were homologous to those in *Arabidopsis* and *Oryza*. Protein structure analysis revealed that different subfamilies contained different motifs, but all of them contained Motif 2. Secondary and tertiary protein structure analysis indicated that the *CsNF-YB* and *CsNF-YC* subfamilies had a high content of alpha helix structures. *Cis*-element analysis showed that elements related to drought stress were mainly concentrated in the *CsNF-YB* and *CsNF-YC* subfamilies, with *CsNF-YB3* and *CsNF-YC12* having the highest content. The results of a transcriptome analysis showed that there was a trend of downregulation of almost all *CsNF-Ys* in leaves under drought stress, while in roots, most members of the *CsNF-YB* subfamily showed a trend of upregulation. Additionally, seven genes were selected for real-time reverse transcription quantitative PCR (qRT-PCR) experiments. The results were generally consistent with those of the transcriptome analysis. The regulatory roles of *CsNF-YB 1*, *2*, and *4* were particularly evident in the roots. The findings of our study may make a great contribution to the understanding of the role of *CsNF-Ys* in stress-related metabolic processes.

## 1. Introduction

Transcription factors have a significant role in almost all developmental processes in plants. *NUCLEAR FACTOR Y (NF-Y)* represents a group of sequence-specific transcription factors that are located in the cell nucleus and bind to the CCAAT element in gene promoters [1]. *NF-Y* is a heterotrimeric complex composed of three different subunits: *NF-YA*, *NF-YB*, and *NF-YC*. All three subunits possess conserved DNA-binding domains and subunit interaction domains [2]. In the formation of the heterotrimeric complex, *NF-YB* and *NF-YC*, which have H2B and H2A structural domains, form a tight histone dimer in the cytoplasm; then, the dimer translocates into the nucleus and interacts with *NF-YA* to form the final heterotrimer. The CCAAT element is one of the most common *cis*-elements in eukaryotic promoters [3]. Due to the specific binding of *NF-YA* to the CCAAT box, the heterotrimer functions as a transcription factor that regulates downstream genes containing CCAAT binding sites in the promoter region [4]. *NF-Y* transcription factors have been extensively studied in plants.

In some plants, *AtNF-Ys* are involved in the regulation of seed morphology [5], plant bud and root differentiation [6,7], flowering time [8,9], and chlorophyll synthesis [10]. Additionally, *AtNF-Y* plays important roles in responding to abiotic stresses, such as drought [11,12], salt [13], cold [14], and heat [15]. *OsNF-YC*5 negatively regulates the salt tolerance of *Oryza sativa* in response to abiotic stress, specifically salt stress [16]. Overexpression of *GbNF-YA6* significantly induced the expression of heat shock factors (*GbHSFs*) in callus tissue under heat stress, suggesting that *GbNF-YA6* can effectively enhance plant heat tolerance [17]. The *PhNF-Y* gene family—especially the *PhNF-YA* and *PhNF-YB* subfamilies—exhibited a comprehensive response to cold, heat, drought, and salt stress [18]. Recent studies have investigated the *NF-Y* gene family in corn [19], Chinese cabbage [20], melon [21], peach [2], and Phalaenopsis orchid [22], demonstrating their significance in physiological ecology and abiotic stress responses across a variety of species.

The orchid family is one of the largest and most widely distributed families in the plant kingdom, with over 28,000 species [23]. Recently, genomic sequencing was conducted for various *Cymbidium* species, providing valuable resources for our study [24,25], which helps in the systematic exploration of the *NF-Y* gene family in the orchid family. *Cymbidium sinense* is a plant species in the orchid family that has a high ornamental value. Its unique deep purple color makes it highly distinctive, and it blooms around the traditional Chinese Spring Festival. With global climate change, arid regions in China are gradually expanding, greatly impacting the environments in which orchids can survive and be cultivated. Drought stress is one of the bottleneck factors for plant growth and development [26,27]. In epiphytic orchids, water stress is the most important abiotic factor limiting the growth and development of epiphytic orchids [28]. Although *C. sinense* belongs to terrestrial orchids, its high ornamental and economic value makes the cultivation of drought-tolerant varieties of significant importance for the conservation of orchid germplasm resources and improvement in the orchid industry supply chain. Although *NF-Y* has been widely reported in model plants and major crops, its roles and functions in the floral development of orchid species are not well understood. This study investigated the response of the *NF-Y* gene to drought stress in *C. sinense* by using bioinformatics methods and provides a set of potential drought-resistant candidate genes. It is of great significance for the identifying of key regulatory factors for drought resistance in *C. sinense*.

## 2. Result

### 2.1. Identification and Physicochemical Properties

**Figure 1:**
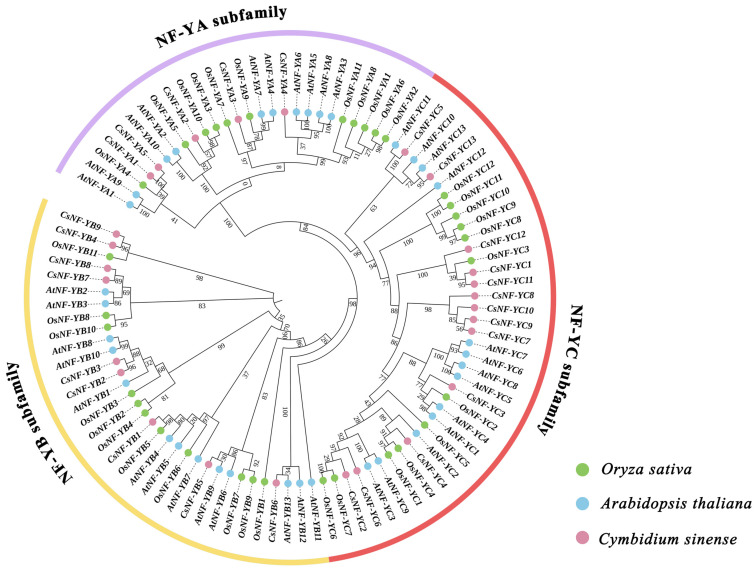
The NJ phylogenetic tree of NF-Y proteins from *C. sinense*, *A. thaliana*, and *O. sativa*. Proteins from *C. sinense* are indicated by pink dots, proteins from *A. thaliana* are indicated by blue dots, and proteins from *O. sativa* are indicated by green dots. The purple, yellow, and red bands indicate the *NF-Y*A, *NF-Y*B, and *NF-Y*C subfamilies, respectively.

All 27 of the *CsNF-Y*s were identified (Table S1) and are presented in a phylogenetic tree (Figure 1). To explore the phylogenetic relationship of *CsNF-Y*s in *C. sinense*, a neighbor-joining (NJ) phylogenetic tree was performed using 27 *CsNF-Y*s and 36 *AtNF-Y*s. Based on the classification of *NF-Y* gene families in *A. thaliana* and the composition of conserved protein domains in *CsNF-Y*s, the 27 *CsNF-Ys* were divided into three subfamilies: *CsNF-YA*, *CsNF-YB*, and *CsNF-YC*. Among these subfamilies, *CsNF-YA* had the fewest members, as it had only 5, while *CsNF-YC* had the most members with 13 genes. Additionally, the *CsNF-YB* subfamily consisted of nine members. We presented the evolutionary distances between different proteins in Figure S1.

The results of the analysis of the protein physicochemical properties in *CsNF-Ys* revealed that the isoelectric point (pI) of the NF-Y proteins in *C. sinense* ranged from 4.63 (*CsNF-YB6*) to 9.95 (*CsNF-YA3*) (Table 1). The molecular weight ranged from 12.14 kDa (*CsNF-YC11*) to 32.87 kDa (*CsNF-YA5*). The number of amino acids ranged from 112 (*CsNF-YC11*) to 293 (*CsNF-YA5*), and the aliphatic index ranged from 48.15 to 105.8. The protein physicochemical properties showed that the average hydropathy index (GRAVY) of the CsNF-Y proteins was mostly negative, with a minimum of −0.805 (*CsNF-YA5*). Notably, *CsNF-YC11* stood out with a positive hydropathy index of 0.014. Therefore, the CsNF-Y proteins were predominantly hydrophilic, albeit with varying degrees of hydrophilicity. Among these 27 CsNF-Y proteins, only *CsNF-YB1* and *CsNF-YC5* had instability coefficients that were less than 40, and those of the majority of the CsNF-Y proteins were greater than 50 (63%).

### 2.2. Gene Conserved Motif Analysis

**Figure 2:**
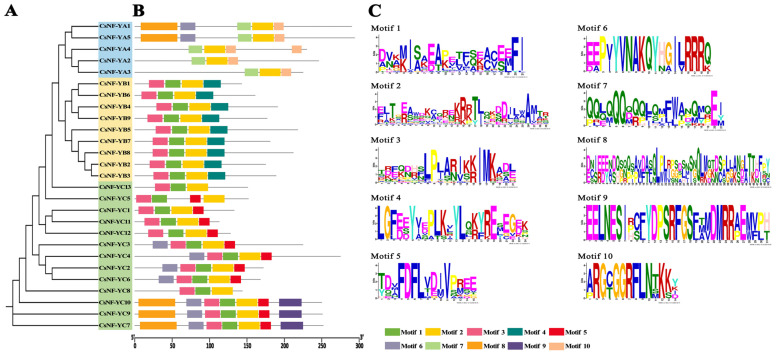
The phylogenetic tree and architecture of conserved protein motifs of the *CsNF-Y* gene family in *C. sinense*. (**A**) The phylogenetic relationship of CsNF-Y proteins in *C. sinense*. Members of the *CsNF-YA*, *CsNF-YB*, and *CsNF-YC* subfamilies are indicated by blue, yellow, and green colors, respectively. (**B**) The motif patterns of CsNF-Y proteins. Different colors represent different motifs, which are numbered from 1 to 10. Protein lengths can be estimated based on the scale at the bottom right. (**C**) The sequence information for Motifs 1–10, respectively. Protein lengths can be estimated based on the scale at the bottom.

The conserved motifs of the CsNF-Y proteins were further characterized with the MEME software. The results revealed a total of ten conserved motifs (Figure 2). Among them, Motif 2 was detected in all 27 genes, while Motif 9 was only detected in 3 genes. In the members of the *CsNF-YA* subfamily, Motifs 2, 6, 7, 8, and 10 were detected, with Motifs 2, 7, and 10 being present in all members of the *CsNF-YA* subfamily. Motifs 6 and 8 were detected in *CsNF-YA1* and *CsNF-Y*A5. In the *CsNF-YB* subfamily, Motifs 1, 2, 3, and 4 were detected, with Motif 4 being the only shared motif. In the *CsNF-YC* subfamily, Motifs 1, 2, 3, 5, 6, 8, and 9 were detected. Motif 5 was detected in almost all members of the subfamily, while Motif 6 was present in *CsNF-YC2*, *3*, *4*, *6*, *7*, *9*, and *10*. Motifs 8 and 9 were only found in *CsNF-YC7*, *9*, and *10*. All motif logos are displayed in Figure 2C.

### 2.3. Analysis of Amino Acid Conserved Domains

**Figure 3:**
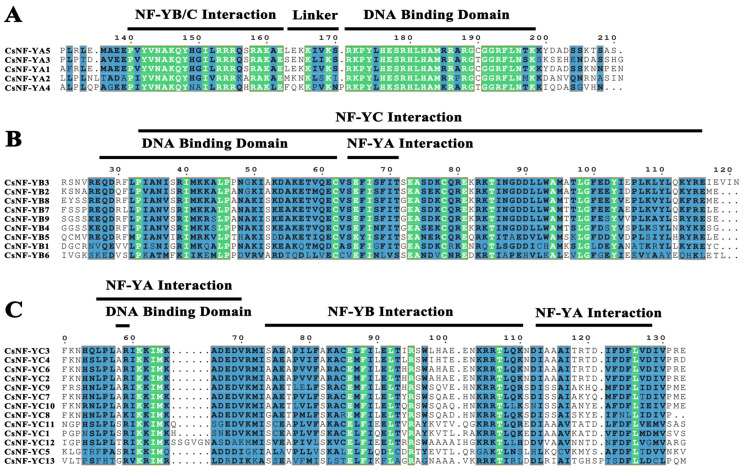
The multiple-sequence alignment of CsNF-Y protein sequences. (**A**–**C**) The sequence alignment of highly conserved domains of CsNF-YA, CsNF-YB, and CsNF-YC proteins in *C. sinense*.

The protein sequences of CsNF-YA, CsNF-YB, and CsNF-YC were subjected to multiple-sequence alignment analysis by using Phylosuite and ESPript 3.0 to identify and analyze the conserved domains of the CsNF-Y proteins. Each member of these three subfamilies contained a highly conserved DNA-binding domain, and the DNA-binding and subunit interaction domains were represented. The multiple-sequence alignment of the CsNF-YA proteins revealed a conserved region consisting of approximately 60 amino acids (Figure 3A). Similarly, the protein sequence alignments of CsNF-YB (approximately 90 amino acids) and CsNF-YC (approximately 75 amino acids) also identified two conserved regions each (Figure 3B,C).

### 2.4. Gene Structure and Characterization Analysis

**Figure 4:**
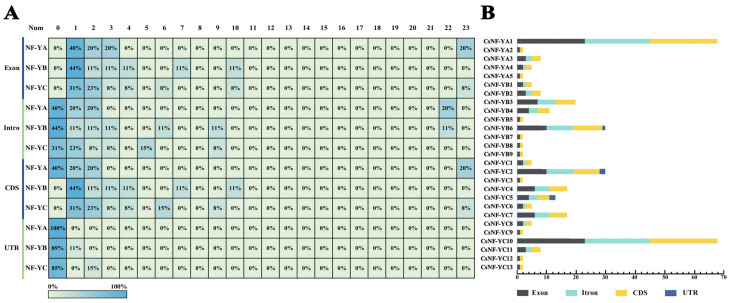
The numbers of exons, introns, coding sequences (CDSs), and untranslated regions (UTRs) in the *CsNF-Y* genes. (**A**) The proportion of members with different numbers of introns, exons, CDSs, and UTRs in each subfamily among the total subfamily members. Green indicates a low proportion, and blue indicates a high proportion. (**B**) The numbers of exons, introns, CDSs, and UTRs on each gene in the *CsNF-Y* gene family, with different colors indicating different structures. The quantity can be estimated based on the bottom axis.

Furthermore, with the aim of enhancing our understanding of the structural composition of genes, the genomic DNA sequences of the *CsNF-Y* genes were analyzed to compare the structures and quantities of introns, exons, coding regions, and non-coding regions (Figure 4). Most members of the *CsNF-Y* family either lacked introns or had only a few introns, with only five genes having more than five introns. Additionally, the number of introns in the members of the *CsNF-YB* subfamily were consistently less than 10, while the *CsNF-YA* and *CsNF-YC* subfamilies each had one gene with 22 introns. Interestingly, the *CsNF-YA1* and *CsNF-YC10* genes, which had 22 introns, also had the highest number of exons [23].

### 2.5. Chromosome Distribution Analysis

**Figure 5:**
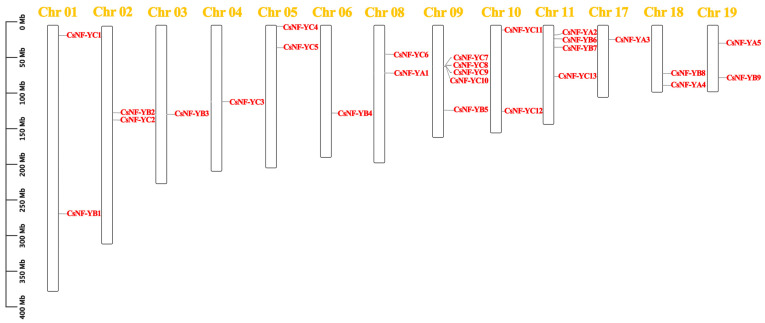
Chromosome distribution of NF-Y gene family of *C. sinense.*

In order to investigate the chromosomal distribution of the *CsNF-Y* genes, we analyzed the genome of *C. sinense*. The analysis revealed an uneven distribution of a total of 27 NF-Y genes across 13 chromosomes (Figure 5). Based on the subfamily classification and chromosome position information, we assigned names to these genes: *CsNF-YA1*–*5*, *CsNF-YB1*–*9*, and *CsNF-YC1*–*13*. The highest number of *NF-Y* transcription factors (5, 18.5%) was found on chromosome 9. Chromosome 11 contained four *CsNF-Y* genes (14.8%), while chromosomes 3, 4, 6, and 17 each had only one *CsNF-Y* gene.

The *CsNF-YC* subfamily was distributed on most chromosomes, while the *CsNF-YA* subfamily was found on chromosomes 8, 11, 17, 18, and 19. Chromosome 17 exclusively contained a member of the *CsNF-YA* subfamily, Cs*NF-Y*A*3*. The *CsNF-YB* subfamily was distributed on chromosomes 1, 2, 3, 6, 9, 11, 18, and 19, with chromosomes 3 and 6 only harboring members of the *CsNF-YB* subfamily. The members of the *NF-YC* subfamily were widely distributed, with chromosomes 4, 5, and 10 exclusively containing members of the *CsNF-YC* subfamily, and no members of the *CsNF-YC* subfamily were found on chromosomes 3, 6, 17, 18, and 19. Some *CsNF-Y*C transcription factors with similar conserved structures were located on the same chromosome, such as the four members of the *CsNF-Y*C subfamily and *CsNF-YB5* on chromosome 9.

### 2.6. cis-Elements Analysis

**Figure 6:**
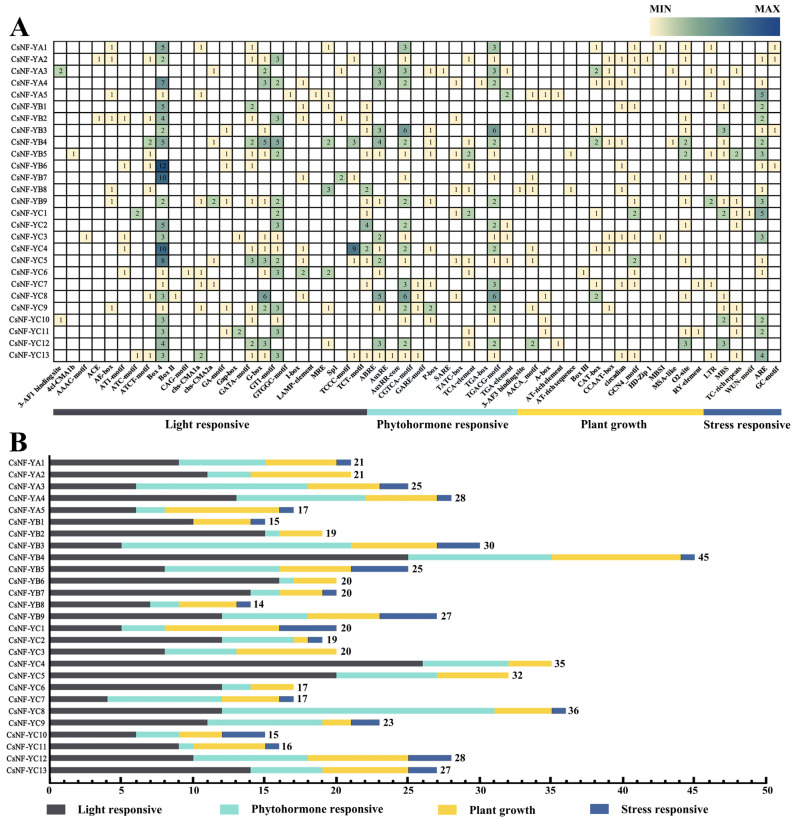
Analysis of *cis*-regulatory elements in *CsNF-Ys*. (**A**) Heatmap of the quantity of *cis*-elements. The color bar above and the values in the boxes represent the classification and quantity of *cis*-elements, with deep blue in the boxes indicating a high quantity and yellow indicating a low quantity. Below are four different color bars: black, yellow, light blue, and deep blue, representing *cis*-elements for light response, plant hormone response, stress response, and plant growth and development, respectively. (**B**) The sum of the four types of *cis*-elements in each gene is represented by a bar chart with different colors, with each color indicating a different response element. The total number of *cis*-elements on each gene was annotated at the end of each bar chart.

To speculate on the potential functions of *CsNF-Y* genes, an analysis of the prediction of *cis*-elements was conducted by using the promoters of these genes (Figure 6). Several categories of *cis*-elements were observed in the *CsNF-Y* genes, and they were roughly classified into four types: light-responsive elements, plant growth elements, stress-responsive elements, and hormone-responsive elements.

Light-responsive elements (306 in total) were the most abundant *cis*-elements found in the promoters of the *CsNF-Y* genes. Hormone-responsive elements (159 in total), including methyl jasmonate (MeJA, 84 elements), salicylic acid (SA, 13 elements), auxin (10 elements), gibberellin (21 elements), and abscisic acid (31 elements), were detected in *CsNF-Y* gene promoters. Additionally, the promoters included stress-related elements associated with anaerobic response (44 elements), drought response (17 elements), defense and stress response (10 elements), low-temperature response (9 elements), and wound response (1 element). Furthermore, promoter elements that were related to metabolism (18 elements), endosperm expression (15 elements), circadian rhythm control (9 elements), and cell cycle regulation (2 elements) were also detected.

### 2.7. Analysis of the Interaction Network and Secondary and Tertiary Structures of CsNF-Ys Proteins

**Figure 7:**
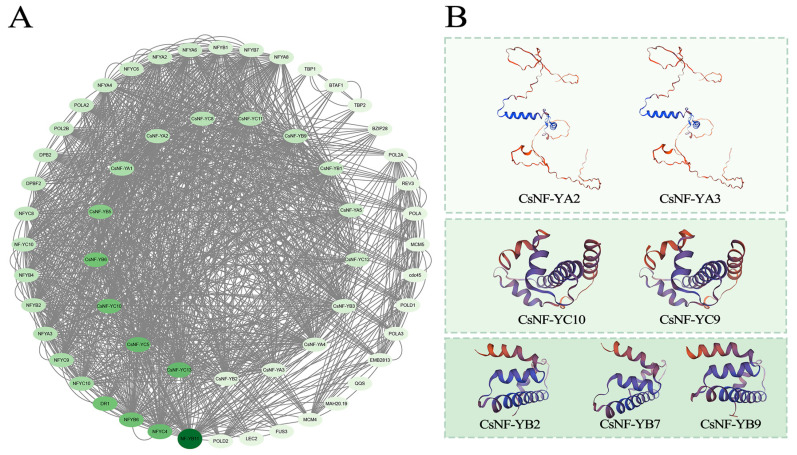
The predicted protein–protein interaction network and protein tertiary structures of CsNF-Ys. (**A**) The predicted protein–protein interaction network of CsNF-Ys. Each green oval represents a protein, and the darker the green, the stronger the interaction between the proteins. The outer circle represents proteins interacting with CsNF-Ys, while the inner circle represents NF-Y proteins of *C. sinense*. The interactions between these proteins are indicated by gray lines. (**B**) The predicted protein tertiary structures of CsNF-Ys.

Protein–protein interaction prediction was conducted to further understand the biological function and regulatory network of CsNF-Ys. A total of 57 protein–protein interactions were discovered, and apart from 17 CsNF-Y proteins, a total of 40 proteins interacted with CsNF-Ys (Figure 7A). The proteins interacting with CsNF-Ys have been functionally validated to be associated with drought stress, such as through the *DR1* gene [29] and the *BZIP* gene family [30]. Moreover, proteins such as QQS, which were involved in plant development or the response to abiotic stress [31,32], were predicted to interact with CsNF-Ys.

The analysis of the secondary structure of the CsNF-Y proteins (Table S2) revealed that the alpha helix occupied the largest proportion of the protein structure, followed by the random coil and then the extended strand, with the smallest proportion belonging to the beta-turn. The proportion of alpha helix structures in the CsNF-YA and CsNF-YC subfamilies was significantly larger than the proportion of random coil structures, while this difference was less pronounced in CsNF-YB. The tertiary structure of the CsNF-Y proteins was predicted through an online analysis with SWISS-MODEL, and the results are displayed in Figure 7B.

### 2.8. Analysis of Expression Patterns under Drought Stress in Leaves and Roots

**Figure 8:**
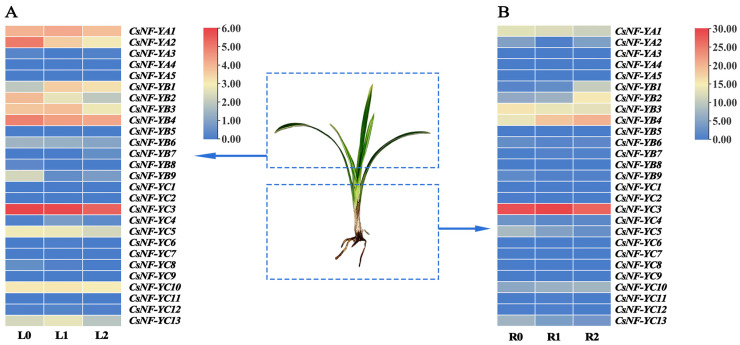
The expression profiles of *CsNF-Y* genes under different levels of drought stress in leaves and roots. (**A**) L0, L1, and L2 represent the control group, mild drought treatment, and severe drought treatment in leaves, respectively. (**B**) R0, R1, and R2 represent the control group, mild drought treatment, and severe drought treatment in roots, respectively. The color bar below represents the normalized FPKM values: red, high expression level; yellow, low expression level; blue, no expression. The detailed FPKM values are listed in Table S3.

By comparing the FPKM values of leaves and roots under three levels of drought stress, we detected the expression profiles of individual *CsNF-Y* genes to investigate the organ-specific expression patterns of the *NF-Y* gene family in *C. sinense* under different drought conditions (Figure 8).

In leaves, the FPKM values (FPKM > 5) in 10 out of 27 *CsNF-Y* genes were significant (Table S3a). A comparison was made between L0 and L1, where eight genes were upregulated and nine were downregulated, while between L1 and L2, three genes were upregulated and fifteen were downregulated. Seven genes—*CsNF-YA2*, *CsNF-YB2*, *CsNF-YB4*, *CsNF-YB6*, *CsNF-YC3*, *CsNF-YC5*, and *CsNF-YC8*—were downregulated under both mild (L1) and severe (L2) drought stress. Notably, except for *CsNF-YB1*, none of the *CsNF-Y* members were upregulated at both drought levels.

In roots, the FPKM values (FPKM > 5) in 9 out of 27 *CsNF-Y* genes were significant (Table S3b). A comparison was made between R0 and R1, where eight genes exhibited upregulation, whereas nine genes showed downregulation. Similarly, between R1 and R2, eight genes were upregulated, while eight were downregulated. Four genes—*CsNF-YB1*, *CsNF-YB2*, *CsNF-YB4*, and *CsNF-YC10*—were upregulated under both mild (R1) and severe (R2) drought stress. In contrast, *CsNF-YA1*, *CsNF-YB3*, *CsNF-YC5*, and *CsNF-YC13* were downregulated under both conditions. 

Among the 27 *CsNF-Y* genes, most of the detected *CsNF-Y* genes (18/27) exhibited altered regulation under drought stress. Notably, *CsNF-B9* and *CsNF-C5* showed consistent expression trends in both roots and leaves across the drought levels. Intriguingly, many members in both organs showed a positive drought response through significant downregulation. No *CsNF-Ys* in the leaves continued their upregulation under both mild and severe drought stress; on the contrary, five *CsNF-Ys* in the roots were concurrently upregulated under both conditions, potentially indicating that there was a more active drought stress response in the roots through transcriptional activation. 

### 2.9. Expression of CsNY-Fs in Response to Drought Stress 

**Figure 9:**
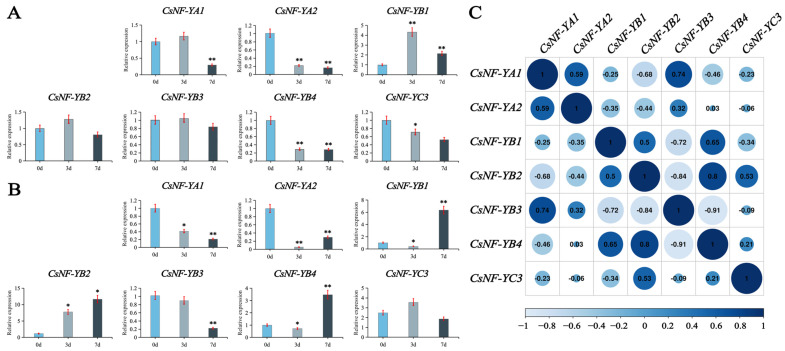
Verification of the effect of *CsNF-Ys* during drought stress with real-time reverse transcription quantitative PCR (qRT-PCR). (**A**) The relative expression levels of each gene in leaves. (**B**) The relative expression levels of each gene in roots. The blue bars represent the relative expression during the control period, indicating no drought stress. The light blue bars represent the relative expression during mild drought stress, and the deep blue bars represent the relative expression during severe drought stress. Black asterisks indicate the *p* values from the significance test (* *p* < 0.05, ** *p* < 0.01). (**C**) The correlation analysis for these seven *CsNF-Ys* genes. Primers for qRT-PCR are shown in Table S4.

To validate the reliability of the transcriptome data of *CsNF-Y* genes in leaves and roots during drought stress, we selected seven genes (*CsNF-YA1*, *CsNF-YA2*, *CsNF-YB1*, *CsNF-YB2*, *CsNF-YB3*, *CsNF-YB4*, and *CsNF-YC3*) with high expression or significant changes based on the transcriptome data and conducted qRT-PCR experiments (Figure 9). The results showed that the expression levels of these seven genes varied during drought stress, and the trends were generally consistent with the changes observed in the transcriptome data.

Consistent with the trend of the transcriptome data, as the severity of drought stress increased, most genes in both leaves and roots showed a negative response. Members of the *CsNF-YB* subfamily in the root exhibited a positive response, and their expression levels increased with the deepening of drought stress, consistent with the trend observed in the transcriptome data. Further correlation analysis revealed a highly significant correlation among *CsNF-YA1, CsNF-YB1, CsNF-YB2, CsNF-YB3,* and *CsNF-YB4* (Figure 9C). *CsNF-YB2* showed a significant correlation with *CsNF-YB4*, indicating a possible association in the expression of these two genes.

## 3. Discussion

The *NF-Y* transcription factor is crucial for plant growth and development at various stages. Abiotic stresses (temperature, salinity, and drought) significantly impact plant growth. In response to these stresses, plants have developed strategies for enhancing stress tolerance. *NF-Y* transcription factors have attracted considerable attention in the field of plant research due to their essential functions in plant–microbe interactions, root development, and adaptation to water-related stress [33]. In plants, multiple genes encode each subunit of *NF-Y*, and the number of genes involved may vary among different species [1]. In *A. thaliana*, 36 *NF-Y* genes have been identified. Among them, the *AtNF-YA* subfamily consists of 10 members, while the *AtNF-YB* and *AtNF-YC* subfamilies each have 13 members [34]. A total of 34 *NF-Y* genes were identified in *O. sativa*, with 11 members of *OsNF-Y*A, 11 members of *OsNF-Y*B, and 12 members of *OsNF-Y*C [32]. In this study, we identified 27 *CsNF-Y* genes: 5 *CsNF-A*, 9 *CsNF-YB*, and 13 *CsNF-YC* genes. The variations in family size across species suggests evolutionary changes influenced by factors such as the living environment.

Phylogenetic analysis indicated a close relationship between *CsNF-Y a*nd *AtNF-Y* in the phylogenetic tree. *C. sinense* divides *CsNF-Y* into three subgroups based on *AtNF-Y*, and functionally similar genes are grouped together. One can predict the functions of *NF-Ys* based on the known functions of *AtNF-Ys* [35]. Earlier studies found that *AtNF-YA1* and *AtNF-YA9*, which are located on the same branch, are crucial regulators of embryonic development and flowering time [7,36]. This suggests that the *CsNF-YA1* and *CsNF-YA5* genes, which are on the same branch in the phylogenetic tree, may have similar functions. *NF-YB3* from *Picea wilsonii*, which is transiently expressed in *Arabidopsis*, exhibited characteristics of salt and drought stress tolerance. In Phalaenopsis, the *PhNF-YC7* gene and other *NF-Y* family genes may indirectly regulate plant development and flowering time in response to low temperatures. We speculate that the *NF-YC* subfamily in *Cymbidium* may also have similar functional responses [22]. 

Multiple-sequence alignment analysis showed that the best CsNF-Y proteins contained conserved regions as a result of subunit interactions and DNA binding (Figure 4). These regions are also present in other plants [37]. Previous studies indicate that the DNA-binding structure of *NF-Y* can bind to the CCAAT site [38]. In addition, conservative motif analysis of the 27 CsNF-Y proteins revealed that all members contained Motif 2. Interestingly, all members of the *CsNF-YB* subfamily had acquired Motif 4, which was not present in the other subfamilies. The reliability of the phylogenetic tree and clustering was further confirmed by the presence of a similar gene structure composition and conserved motifs within specific subfamilies.

Consistent with the prediction of the *cis*-elements of the *NF-Y* family in other species, *CsNF-Ys* contained 39 *cis*-elements. The *CsNF-Y* genes were regulated by *cis*-elements and played crucial roles in the stress response. Genes containing the drought-responsive element MBS (MYB binding site) were mainly members of the *CsNF-YB* and *CsNF-YC* subfamilies. The member *CsNF-YB3* in the *CsNF-YB* subfamily, which contains the most MBS *cis*-elements, showed consistent expression in both leaves and roots, with the transcriptome changes following a similar trend. However, the gene *CsNF-YC12*, which contains the most MBS *cis*-elements in the *CsNF-YC* subfamily, was not expressed in either the leaves or roots. Instead, genes *CsNF-YC5* and *CsNF-YC10*, which contain fewer MBS *cis*-elements, were expressed in both leaves and roots with consistent expression patterns. Combined with the transcriptome heatmap, it was found that the expression levels of most *CsNF-YB* subfamily members in the root system gradually increased with the severity of drought stress. In contrast, most *CsNF-YA* and *CsNF-YC* subfamily members showed lower expression levels under severe drought compared to the control group. This suggested that the response of *CsNF-YB* to drought stress may be closely related not only to MBS but also to Motif 4. Intron–exon structure analysis indicated that most *CsNF-Y* sequences (37%) consisted of 0 introns and 1 exon, while *CsNF-YA1* and *CsNF-YC10* contained 22 introns and 23 exons. This situation was rarely observed in the *NF-Y* family in other species. In *Citrullus lanatus*, an analysis of the gene structure of *ClNF-Y* exhibited that all genes consisted of 0–5 introns and 1–6 exons [39]. The structural analysis of *MaNF-Y* genes indicated that all of them contained 0–4 introns, and they evidenced a relatively consistent intron–exon organization [40]. The high diversity in gene structure suggests extensive differentiation during the formation and evolution of the *CsNF-Y* genome. 

We also investigated the expression patterns of *CsNF-Ys* under varying levels of drought stress and in different tissues to understand their roles in the drought stress response in *C. sinense*. In addition, under severe drought stress (L2 and R2), compared to the control group (L0 and R0), most of the *CsNF-Y* genes were upregulated in the roots and downregulated in the leaves. Some *TaNF-Y* genes in wheat leaves exhibited a significant upregulation in response to drought stress—particularly *TaNF-YB2* [41]. Interestingly, in *C. sinense*, slight drought treatment caused a significant change in gene expression in the leaves, while severe drought treatment induced a significant change in gene expression in the roots. We also observed that as the severity of drought stress deepened, most genes in the leaves showed a trend of downregulation; the genes in the *CsNF-YA* and *CsNF-YC* subfamilies were mostly downregulated in the roots, while the genes in the *CsNF-YB* subfamily were upregulated. This result may imply that members of the *CsNF-YB* family with a specific Motif 4 binding structure have some particular stress response mechanisms.

These findings are crucial for identifying potential genes that enhance molecular breeding efficiency. Moreover, these discoveries contribute to the selection of varieties of *C. sinense* with strong resistance to adversity and can provide important reference value for the understanding of the action of *NF-Y* transcription factors under stress conditions. These results may contribute to the improvement in the tolerance of Cymbidium to abiotic stress. 

## 4. Materials and Methods

### 4.1. Identification and Classification of the NF-Y Gene Family in C. sinense

The full-genome data of *C. sinense* were obtained from an article by Yang et al. [25] (NCBI: PRJNA743748, 2021). The NF-Y protein sequences of *A. thaliana* were derived from previous reports [34]. By utilizing the BLASTP function of TBtools-II (version v2.207) [42], a search was conducted in the *C. sinense* genome (e-value 1 × 10^−5^) with *AtNF-Ys* as the reference sequences. The conserved *NF-Y* domain of the sequence was obtained by performing sequence alignment using the BLASTP function on the NCBI-CDD website (https://www.ncbi.nlm.nih.gov/Structure/bwrpsb/bwrpsb.cgi, accessed on 14 November 2023) and Phylosuite (version 1.2.3) [43]. Eventually, genes that did not contain the conserved *NFY* domain were removed, resulting in a final set of 27 *CsNF-Y* genes.

### 4.2. Multiple-Sequence Alignment and Phylogenetic Analysis

The Mafft function in Phylosuite (version 1.2.3) was utilized to perform multiple-sequence alignment of *CsNF-Ys.* The aligned protein sequences were then visualized by using ESPript 3.0 (https://espript.ibcp.fr/ESPript/cgi-bin/ESPript.cgi, accessed on 19 November 2023) with the default parameters. The protein sequences of rice *NF-Ys* were derived from previous reports [44]. Phylogenetic analysis of *NF-Ys* from *C. sinense*, *A. thaliana*, and *O. sativa* was conducted by using Phylosuite (version 1.2.3) (neighbor-joining algorithm with 1000 bootstrap replicates). The resulting phylogenetic tree was visualized by using the iTOL website (https://itol.embl.de/personal_page.cgi, accessed on 18 November 2023). Based on the classification method used for *A. thaliana*, the results from Multiple Em for Motif Elicitation (MEME, version 5.5.4, https://meme-suite.org/meme/tools/meme, accessed on 18 November 2023), and the phylogenetic analysis, the *CsNF-Y* gene family was further categorized into three subfamilies: *CsNF-YA*, *CsNF-YB*, and *CsNF-YC*.

### 4.3. Analysis of Protein Physicochemical Properties and Structure of CsNF-Ys

The protein physicochemical properties of *CsNF-Y*s were calculated by using the Protein Parameter Calc function in TBtools-II (version v2.207). MEME (version 5.5.4, https://meme-suite.org/meme/tools/meme, accessed on 12 December 2023) was employed to analyze the conserved motifs. The numbers of exons, introns, CDSs, and UTRs of the *CsNF-Y* genes were obtained from the genome annotation file. The above analyses were visualized by using TBtools-II (version v2.207) and Excel 2021. The protein–protein interaction network predictive analysis used the STRING database (version 12.0, https://cn.string-db.org, accessed on 19 November 2023) with no more than 20 interactors as references to Arabidopsis homolog. Protein secondary structure prediction was performed using SOPMA (https://npsa-pbil.ibcp.fr/cgi-bin/npsa_automat.pl?page=npsa_sopma.html, accessed on 19 November 2023). Protein tertiary structure prediction was conducted using SWISS-Model (https://swissmodel.expasy.org/interactive, accessed on 19 November 2023). The default parameters were used for all the structure prediction methods mentioned above.

### 4.4. Chromosome Distribution and cis-Elements in the Promoters of CsNF-Ys

Information on the genes’ distribution on the chromosomes was obtained from the annotation file of the *C. sinense* genome. It was confirmed and visualized by using TBtools-II (version v2.207). The genomic sequences of the promoter regions (2000 bp) upstream of each *CsNF-Y* gene’s start codon were extracted by using Tbtools-II (version v2.207). The *cis*-elements in the promoter regions were predicted and annotated by using the Plant-CARE database (http://bioinformatics.psb.ugent.be/webtools/plantcare/html/, accessed on 27 November 2023). Visualization was accomplished by using Excel 2021.

### 4.5. Plant Drought Stress Treatment

In early December, drought stress treatment was applied to *C. sinense*. The environmental conditions were set to a light intensity of 25 µmol/(m^2^·s), a light/dark cycle of 12 h/12 h, a temperature range of 22–26 °C, and a humidity of 40%. The soil moisture level was monitored daily during the drought stress period. Healthy *C. sinense* plants were selected, and seedlings were grown under well-watered conditions for four days. On the fourth day, control samples L0 and R0 were collected. Irrigation was then stopped to initiate the drought stress treatment. Mild-stress samples (L1 and R1) were collected on the third day after irrigation was stopped, and severe-stress samples (L2 and R2) were collected on the seventh day after irrigation was stopped. Three replicates were collected per group, and leaf samples were taken from young leaves that were shorter than ten centimeters, while root samples were taken from newly formed roots at the base of the plants. The samples were collected in liquid nitrogen and stored at −80 °C until RNA extraction. After a 7-day period of drought stress, there were no apparent physical changes in the plant’s appearance that could be observed with the naked eye, such as leaf wilting or yellowing. Therefore, based on the above observations, the plants remained in a healthy state despite the drought stress. Hence, we believe that the impact of the 7-day drought and sampling on RNA can be considered negligible.

### 4.6. Isolation of RNA, cDNA Preparation, and Expression Analysis

The cetyltrimethylammonium bromide (CTAB) method was employed to isolate total RNA from different tissues. Three replicates were collected per tissue. The reagent kit used for preparing RNA was Vazyme’s FastPure Plant Total RNA Isolation Kit (Polysaccharides&Polyphenolics-rich), and the specific preparation method followed the product manual. The reagent kit used for preparing cDNA was Yeasen’s Hifair AdvanceFast One-step RT-gDNA Digestion SuperMix for qPCR, and the specific preparation method followed the product manual. Subsequently, transcriptome sequencing was performed on the MGI2000 sequencing platform. To ensure data quality, the SOAPnuke v1.4.0 software (BGI-Shenzhen, China) was utilized to eliminate low-quality reads, reads containing adapters, and reads with poly-N sequences (with more than 5% unknown bases) [45]. The resulting clean reads were aligned to the nucleotide sequences of *CsNF-Ys* by using HISAT2 version 2.1.0 (University of Texas Southwestern Medical Center, Dallas, TX, USA) [46]. The expression levels of the *CsNF-Ys* were calculated for each exon isoform by using the FPKM (fragments per kilobase of exon per million fragments mapped) method implemented in RSEM v1.2.8 (University of Wisconsin-Madison, Madison, WI, USA) [47,48]. Finally, TBtools-II (version v2.207) was utilized to visualize the expression profiles.

### 4.7. RNA Extraction and Real-Time Reverse Transcription Quantitative PCR (qRT-PCR) Analysis 

To validate the transcriptome data, we selected seven candidate genes for qRT-PCR to confirm their transcriptomic profiles. A total of three biological replicates were collected from two different tissues in three distinct drought stress periods. The collected samples were immediately frozen in liquid nitrogen and stored at −80 °C until total RNA extraction. The stable actin gene (*Mol 022529*) from the same species was chosen as the reference gene [49]. Primers for the stable actin gene and the seven candidate genes were designed using Primer Premier 5.0 [50], as listed in Table S4. For reverse transcription, 1 µL of RNA was subjected to cDNA using the Hifair AdvanceFast One-Step RT-gDNA Digestion SuperMix for qPCR (Beijing, China). The resulting cDNA from each sample was diluted to a concentration of 10 ng/mL, and 2 µL of the diluted cDNA was used as the template for qRT-PCR. The qRT-PCR experiments were performed on the Applied Biosystems^®^ QuantStudio^®^ 3 Real-Time PCR System (Applied Biosystems, Foster City, CA, USA) with SYBR Green dye. The reaction system was 20 μL, and the reaction program was set as follows: 1 min at 95 °C, followed by 40 cycles of 10 s at 95 °C and 20 s at 60 °C. Fluorescence signals were collected at the end of the annealing and extension step. The product specificity was determined through a melting curve analysis, and the Ct values for each sample were obtained. The expression levels of each gene were normalized to the actin internal control gene, and the relative gene expression levels were calculated by using the 2^−ΔΔCT^ method [51]. The correlation analysis of the seven selected genes for qRT-PCR experiments was performed using CorrPlot with the OmicStudio tool (https://www.omicstudio.cn/tool, accessed on 19 December 2023).

## 5. Conclusions

This study examined the *NF-Y* gene family in *C. sinense*. A total of 27 *CsNF-Ys* were identified in *C. sinense* for the first time, and various aspects, including conserved motifs, exon–intron structures, and secondary/tertiary structures of proteins, were analyzed. The findings revealed a high degree of conservation among *CsNF-Y* genes. The identification of *cis*-elements related to drought response in the promoter region of *CsNF-Ys* helped expand the knowledge of the drought resistance pathway in *C. sinense*. This study explored the performance of CsNFYs in three stages of water deficiency and verified their expression patterns in the leaves and roots. The consistent results showed distinct expression patterns of *CsNF-Ys* in the leaves and roots. Three *CsNF-Ys* (*CsNF-YB1*, *CsNF-YB2*, and *CsNF-YB4*) may be potential drought-resistant candidate genes. This study may provide valuable information for the study of the stress response mechanisms of *NF-Y* genes in different tissues of orchids and other species.

## Figures and Tables

**Table 1:** Protein information of the *NF-Y* gene family in *Cymbidium sinens*e, including the gene ID, gene name, genomic position, and protein physicochemical properties.

Gene ID	Gene Name	Chr NO.	Location	Number of Amino Acids	Molecular Weight (kDa)	Theoretical pI	Instability Index	Aliphatic Index	Grand Average of Hydropathicity
*Mol019234*	*CsNF-YA1*	chr08	67,164,417–67,204,027	289	32.50	8.84	57.99	58.79	−0.78
*Mol027851*	*CsNF-YA2*	chr11	16,254,640–16,255,008	245	27.15	9.75	54.38	64.94	−0.671
*Mol003533*	*CsNF-YA3*	chr17	20,206,566–20,207,862	224	24.47	9.95	69.36	60.58	−0.771
*Mol028384*	*CsNF-YA4*	chr18	84,252,997–84,253,640	229	25.37	9.93	47.59	80.92	−0.44
*Mol020046*	*CsNF-YA5*	chr19	25,325,945–25,326,205	293	32.87	9.35	61.94	60.34	−0.805
*Mol015093*	*CsNF-YB1*	chr01	264,776,444–264,779,726	142	16.13	6.3	37.11	72.89	−0.71
*Mol009348*	*CsNF-YB2*	chr02	121,704,186–121,705,915	174	19.34	5.97	56.96	61.84	−0.78
*Mol010155*	*CsNF-YB3*	chr03	124,927,390–124,966,757	188	20.57	5.76	54.6	65.96	−0.549
*Mol022192*	*CsNF-YB4*	chr06	123,326,327–123,331,668	190	20.16	9.04	48.19	51.84	−0.729
*Mol009007*	*CsNF-YB5*	chr09	119,339,357–119,339,539	217	23.72	5.64	43.91	70.14	−0.553
*Mol015140*	*CsNF-YB6*	chr11	21,094,709–21,099,846	160	18.07	4.63	45.77	76.19	−0.527
*Mol020786*	*CsNF-YB7*	chr11	31,574,371–31,574,769	180	19.52	6.22	46.07	58.61	−0.676
*Mol000779*	*CsNF-YB8*	chr18	67,770,568–67,771,008	211	22.16	6.06	47.93	48.15	−0.665
*Mol022232*	*CsNF-YB9*	chr19	73,711,581–73,712,652	176	19.44	4.78	53.9	62.67	−0.61
*Mol021145*	*CsNF-YC1*	chr01	14,533,186–14,533,851	132	14.92	9.73	51.34	90.83	0
*Mol006573*	*CsNF-YC2*	chr02	131,790,300–131,790,867	171	19.53	5.5	52.88	78.3	−0.531
*Mol016977*	*CsNF-YC3*	chr04	107,344,266–107,383,215	224	25.84	9.94	76.21	73.75	−0.559
*Mol010830*	*CsNF-YC4*	chr05	1,569,897–1,578,459	274	30.31	5.61	63.06	72.41	−0.354
*Mol006806*	*CsNF-YC5*	chr05	31,684,723–31,686,067	151	17.02	9.61	37.63	80.07	−0.594
*Mol003568*	*CsNF-YC6*	chr08	40,739,665–40,746,569	167	19.33	5.77	54.89	73.71	−0.617
*Mol006576*	*CsNF-YC7*	chr09	57,558,417–57,571,073	251	28.80	5.57	58.05	76.61	−0.467
*Mol006575*	*CsNF-YC8*	chr09	57,732,435–57,733,510	143	16.34	7.82	42.83	105.8	−0.129
*Mol006574*	*CsNF-YC9*	chr09	57,813,604–57,814,353	250	29.31	5.27	63.8	70.64	−0.672
*Mol006573*	*CsNF-YC10*	chr09	57,884,670–57,925,774	249	28.88	5.38	64.55	83.94	−0.408
*Mol005117*	*CsNF-YC11*	chr10	6,723,581–6,723,919	112	12.14	8.5	52.85	90.62	0.014
*Mol006317*	*CsNF-YC12*	chr10	120,958,331–120,966,041	127	13.72	9.57	44.46	85.98	−0.136
*Mol004335*	*CsNF-YC13*	chr11	72,273,837–72,280,957	150	16.33	9.26	59.39	73.6	−0.406

## Data Availability

The genomic data of *Cymbidium sinense* are derived from NCBI: PRJNA743748. The sequence data of *CsNFYs* used in the study can be found in Table S1. Sequencing-related data to support the findings are available from the authors upon request.

## Supplementary Materials

The following supporting information can be downloaded at: https://www.mdpi.com/article/10.3390/ijms25053031/s1.
